# Surgery—Syphilitic Alterations of the Teeth

**Published:** 1881-09

**Authors:** 


### SURGERY.

### SYPHILITIC ALTERATIONS OF THE TEETH.

M. Parrot has devoted several lectures to the description of these alterations, described by some authors as of a rachitic nature, whilst the author regards them as one of the products of hereditary syphilis. The following is a brief outline of the argument used by him to enforce his position: The typical alteration is found in the first molar, where it is constant if existing in other parts of the mouth. The altered part seems free in the middle of the teeth, and appears on a lower level than the sound part, from which it is separated by a sort of furrow. It is yellow, of an ochrey aspect; the cuspids are more pointed and filled with small elevations like grains of sand, and extremely friable. On the other hand, the part of the tooth upon which these altered parts rest is normal, often covered as if with a coat of enamel. The peculiarity of this alteration is the sort of retraction which the tooth has undergone at the same time, and which has given rise to the separation between the healthy and affected parts.

The cup-shaped atrophy, next described, may present itself alone or associated with other varieties. It is more particularly observed upon the upper middle incisors. The tooth is generally large and high, and on the anterior and posterior face are observed small depressions of varying numbers—from one to eight. Their diameter does not exceed one millimetre, and they are in a horizontal row, united by small grooves or separated by a fold of enamel. At their level the tooth is not enameled; it is of a dirty yellow, and the dentine is often exposed. This form of atrophy is very remarkable, and seems to be the elementary form of other varieties.

The third form is the sulciform atrophy. It is rarely found in the molars; it occurs chiefly in the incisors, and consists in horizontal furrows in numbers from one to three—rarely four—always horizontal and parallel near the maxillary border. A fourth variety is a hatchet shaped atrophy. This is observed in the first dentition, only in the incisors, and almost always in the superior middle. This alteration is not primitive, being produced after the complete development of the tooth, whilst the other forms described commence when the tooth is still in its sac. The fifth variety is that to which Hutchinson has called attention, consisting of notches, occuring chiefly in the superior middle incisors, but being also found in others.

All of these varieties may be more or less considerably modified, but are almost always accompanied by certain consecutive alterations. First of all is the color, which may be modified, even in very careful persons, becoming yellowish or even greenish. In those exposed to different dusts, these attach themselves to the surface of the teeth, and give rise to various colors. There is also an abundant deposit of tartar. By far the most important consequent alteration is caries. The molars are lost early, the jaw is atrophied, and spaces, as normally seen in horses, are soon established.

There are, however, changes in the teeth which must not be confounded with those described. Thus, at the second dentition, the incisors often present at their free edges saw-like notches; then it may accur that from friction the incisors may cut a bevel-edge on each other. The hatchet-shape may be determined by caries. And, again, there exists normally in some subjects true furrows. It is therefore necessary to be careful in making a diagnosis.—*St. Louis Medical and Surgical Journal.*

